# Neuroprotective Effects of Pomegranate Juice against Parkinson’s Disease and Presence of Ellagitannins-Derived Metabolite—Urolithin A—In the Brain

**DOI:** 10.3390/ijms21010202

**Published:** 2019-12-27

**Authors:** Małgorzata Kujawska, Michael Jourdes, Monika Kurpik, Michał Szulc, Hanna Szaefer, Piotr Chmielarz, Grzegorz Kreiner, Violetta Krajka-Kuźniak, Przemyslaw Łukasz Mikołajczak, Pierre-Louis Teissedre, Jadwiga Jodynis-Liebert

**Affiliations:** 1 Department of Toxicology, Poznan University of Medical Sciences, Dojazd 30, 60-631 Poznań, Poland; m.kurpik@wp.pl (M.K.);; 2 Université de Bordeaux, ISVV, EA 4577, Œnologie, 210 Chemin de Leysotte, F-33140 Villenave d’Ornon, France; 3 INRA, ISVV, USC 1366 INRA, IPB, 210 Chemin de Leysotte, F-33140 Villenave d’Ornon, France; 4 Department of Pharmacology, Poznan University of Medical Sciences, Rokietnicka 5a, 60-806 Poznan, Poland; 5 Department of Pharmaceutical Biochemistry, Poznan University of Medical Sciences, Święcickiego 4, 60-781 Poznań, Poland; 6 Department of Brain Biochemistry, Maj Institute of Pharmacology, Polish Academy of Sciences, Smętna 12, 31-343 Kraków, Poland

**Keywords:** urolithin A, ellagic acid, ellagitannins, pomegranate juice, rotenone, antioxidant enzymes, mitochondrial aldehyde dehydrogenase, neurodegeneration, apoptosis

## Abstract

Pomegranate juice is a rich source of ellagitannins (ETs) believed to contribute to a wide range of pomegranate’s health benefits. While a lot of experimental studies have been devoted to Alzheimer disease and hypoxic-ischemic brain injury, our knowledge of pomegranate’s effects against Parkinson’s disease (PD) is very limited. It is suggested that its neuroprotective effects are mediated by ETs-derived metabolites—urolithins. In this study, we examined the capability of pomegranate juice for protection against PD in a rat model of parkinsonism induced by rotenone. To evaluate its efficiency, assessment of postural instability, visualization of neurodegeneration, determination of oxidative damage to lipids and α-synuclein level, as well as markers of antioxidant defense status, inflammation, and apoptosis, were performed in the midbrain. We also check the presence of plausible active pomegranate ETs-derived metabolite, urolithin A, in the plasma and brain. Our results indicated that pomegranate juice treatment provided neuroprotection as evidenced by the postural stability improvement, enhancement of neuronal survival, its protection against oxidative damage and α-synuclein aggregation, the increase in mitochondrial aldehyde dehydrogenase activity, and maintenance of antiapoptotic Bcl-xL protein at the control level. In addition, we have provided evidence for the distribution of urolithin A to the brain.

## 1. Introduction

Studies on dietary polyphenols suggest their beneficial role against Parkinson’s disease (PD), which is mainly attributed to antioxidant, anti-inflammatory, and anti-apoptotic activity [1]. The current research trends also cover their metabolic derivatives, in particular, bioavailable gut microbiota metabolites, which offer a novel preventive approach for the disease [2].

The pomegranate (*Punica granatum* L.) fruit is a rich source of ellagitannins (ETs) such as punicalagin, punicalin, pedunculagin, gallic and ellagic acid esters of glucose, and ellagic acid (EA) [3], which contribute to antioxidative, anti-inflammatory. and antiapoptotic activity of pomegranate and are believed to play an essential role in its wide range of health benefits. A lot of research on the neuroprotective activity of pomegranate juice and extract has been done. Supplementation with pomegranate juice in the drinking water of pregnant and nursing dams has been demonstrated to protect the neonatal brain in an inflammatory [4] and a hypoxic-ischemic (H-I) models [5,6]. These neuroprotective effects have been shown to be attributed to the inhibition of oxidative stress, and a decrease in the production of proinflammatory cytokines [4] and apoptotic proteins [4,5,6]. In adult male rats, pre-administration with pomegranate extract has provided dose-dependent neuroprotection against cerebral ischemia-reperfusion (I/R) brain injury and DNA damage via antioxidant, anti-inflammatory, and anti-apoptotic action [7]. Pomegranate juice and extracts have also been shown to act neuro-protectively against Alzheimer’s disease (AD) in animal models [8,9,10,11,12,13,14]. In older subjects with age-associated memory complaints, who drank 8 ounces of pomegranate juice for four weeks, a significant improvement in verbal and visual memory as well as an increase in plasma Trolox-equivalent antioxidant capacity was observed. Noteworthily, individuals drinking pomegranate juice represented an increased level of a metabolite of pomegranate ellagitannins—urolithin A glucuronide—in plasma [15]. It is believed that pomegranate’s neuroprotective effects are mediated by urolithins—the colonic microbiota ellagitannins (ETs)-derived metabolites [8]. The capability of the in vivo generated urolithin A and B to reduce the formation of advanced glycation end products have been demonstrated to be involved in the neuroprotective effect of pomegranate [16,17]. Urolithin B has also been indicated to suppress neuroinflammation in the cortex, hippocampus, and substantia nigra (SN) of LPS-injected mouse [18]. There is a rapidly growing body of literature dealing with mechanistic in vitro studies on urolithins’ activities, which may contribute to the overall neuroprotective effects reported for pomegranate. Since mitochondrial impairment and the associated oxidative stress, neuroinflammation, and apoptosis are proposed to be critical processes for neurodegeneration, the inhibition of production of intracellular reactive oxygen species (ROS) [18,19], nitric oxide [18], and pro-inflammatory cytokines [18,20] and the prevention of activation of proapoptotic caspases 3 and 9 [19] caused by urolithins A and B in neuronal cell lines, support their involvement in the neuroprotection.

Despite the considerable effort devoted to the studies on beneficial effects of pomegranate in animal models of AD [9,10,11,12,13,14] and H-I brain injury [6,7,21], there is a gap for research involving experimental models of PD in vivo. To the best of our knowledge, merely two studies referring to this subject have been performed [22,23] and their findings were diverse.

PD is the second most prevalent human neurodegenerative disorder, after AD, which is characterized by motor dysfunction associated with a loss of dopaminergic neurons in the midbrain substantia nigra pars compacta (SNpc) and formation of Lewy bodies, mainly composed of misfolded α-synuclein. Around 95% of diagnosed PD cases are sporadic and are a result of a combination of environmental exposures and genetic susceptibility as well as aging, which is believed to be the predominant risk factor. The pathology of the disease is very complex. Nevertheless, oxidative stress, inflammation, and α-synuclein aggregation, which are tightly linked and interdependent, are regarded to play a crucial role in the neurodegeneration [24,25].

Since the knowledge of pomegranate effects against PD is based on very limited data [22,23], the aim of our study was to evaluate the potential neuroprotective capability of pomegranate juice in a rat model. We triggered a PD-like phenotype in rats by prolonged low-dose rotenone treatment [26]. First, we examined whether administration with pomegranate juice to rats intoxicated with rotenone provided any beneficial effects on postural stability and neuronal survival, and then we assessed the influence of the treatment on antioxidant, inflammatory, and apoptotic markers as well as α-synuclein level in the midbrain. Finally, we extended the research by an additional experiment to examine whether urolithin A is present in the brain after treatment with pomegranate juice and therefore could contribute to the observed effects.

## 2. Results

### 2.1. Bodyweight Gain

**Figure 1:**
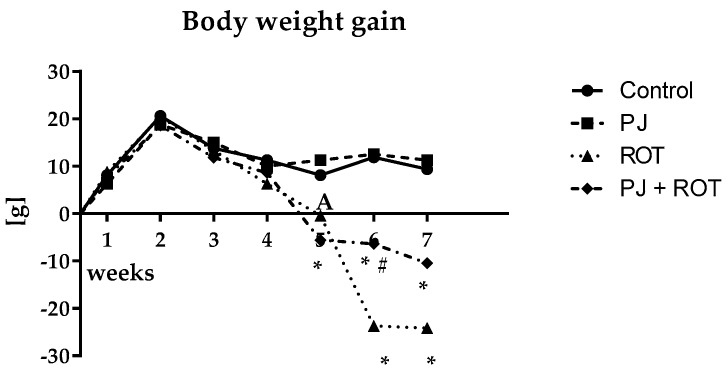
Effect of pomegranate juice treatment (PJ) on body weight gain of rats injected with rotenone (ROT). Data are presented as mean values of eight rats per group and analyzed using one-way analysis of variance (ANOVA) followed by Sidak’s multiple comparisons test. * *p* < 0.05 vs. Control. # *p* < 0.05 vs. ROT.

The mean body weight gain (Figure 1) during the first four weeks was similar across the groups with no statistically significant differences. From the fifth week to the end of experiment, treatment with rotenone alone (ROT) negatively affected body weight gain, which was significantly lower by 105% in week 5 and about three-fold lower in weeks 6 and 7, as compared to the control values. This effect was significantly attenuated, by 57%, in the sixth week of pomegranate juice (PJ) treatment.

### 2.2. Postural Instability

**Figure 2:**
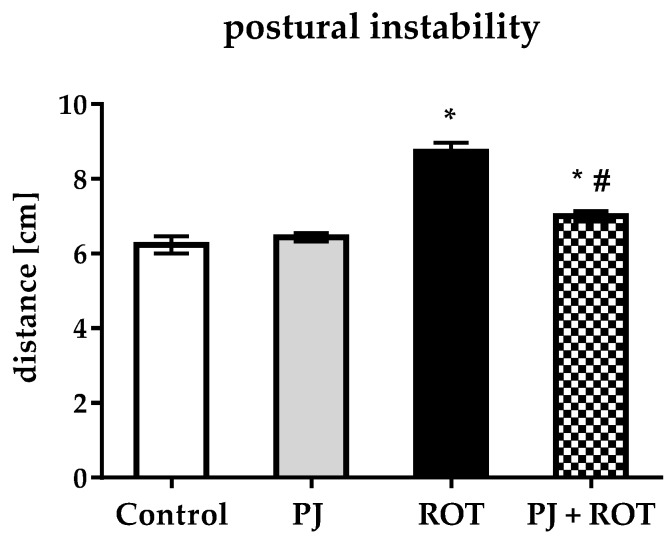
Effect of pomegranate juice treatment (PJ) on postural instability in rotenone (ROT)-injected rats. Data are presented as mean values ±SEM of eight rats per group and analyzed using one-way analysis of variance (ANOVA) followed by Sidak’s multiple comparisons. * *p* < 0.05 vs. Control. # *p* < 0.05 vs. ROT.

To examine whether treatment with PJ could result in behavior improvement, the rats were tested for postural instability (Figure 2). Animals injected with rotenone exhibited statistically significant, 40% greater postural instability as compared to the control. The degree of postural impairment was 20% less in rats that received pomegranate juice and rotenone in combination, as compared to rats injected with ROT alone.

### 2.3. Microscopic Examination

**Figure 3:**
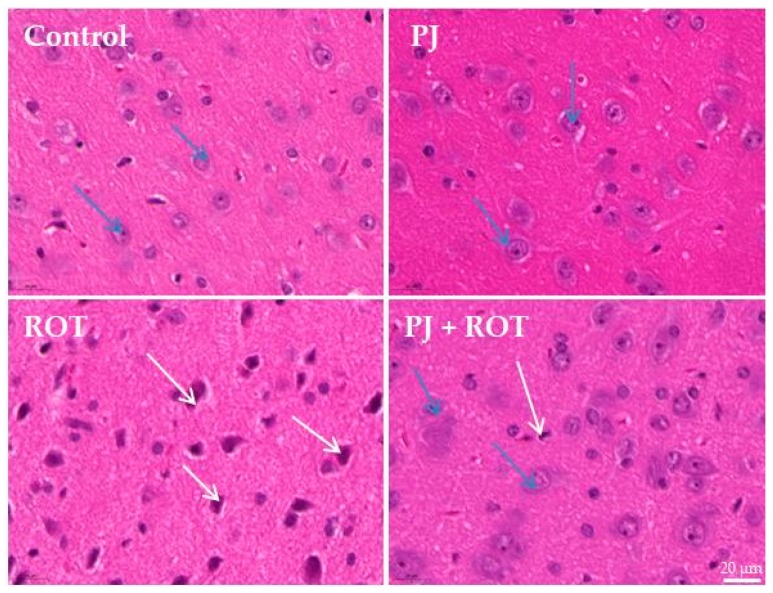
Representative photomicrographs of hematoxylin and eosin (H&E) stained substantia nigra (SN) sections of rats. Control and pomegranate juice alone treated (PJ) rats show normal neurons (blue arrows). Rotenone (ROT) administration caused prominent degeneration of neurons (white arrows). A rat treated with pomegranate juice and rotenone shows normal neurons (blue arrows) and a few cells with signs of degeneration (white arrows). Original magnification ×400; Scale bar—20 μm.

Hematoxylin and eosin (H&E) staining showed marked cell neurodegeneration in SN tissue of rats injected with rotenone (Figure 3). Treatment with pomegranate juice ameliorated the rotenone-induced effect as small deeply stained neurons were only observed. Rats treated with pomegranate juice alone showed normal brain tissue.

### 2.4. Immunofluorescence Staining of TH Positive (TH+) Neurons in the Region of SN

**Figure 4:**
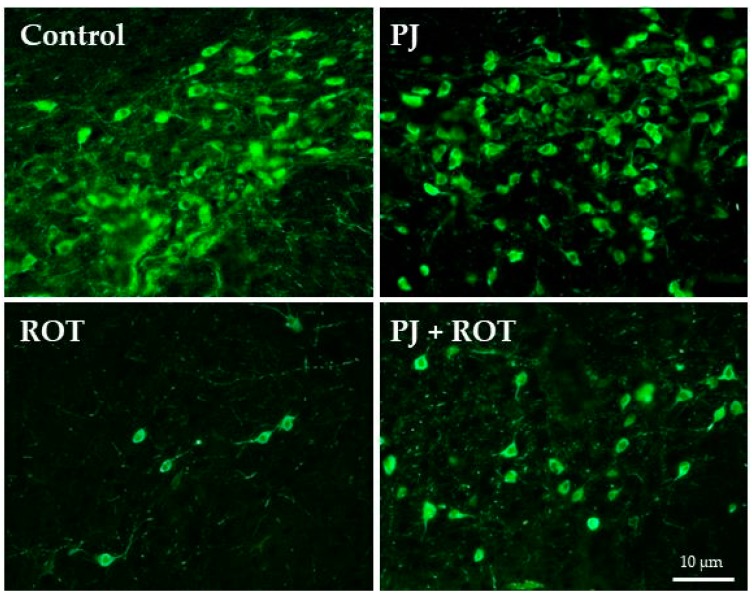
Representative photomicrographs of immunofluorescent staining of TH-positive cells in adjacent microtome sections of substantia nigra (SN) neurons. Rotenone (ROT) administration caused the substantial loss of TH+ neurons, as compared to a control rat (control). Administration of pomegranate juice attenuated this loss (PJ + ROT). The pomegranate juice application (PJ) itself did not cause any effect on TH+ cells survival when compared to control rats. Scale bar—10 μm.

To further examine the suggested beneficial impact of PJ treatment on neurons’ survival, we performed immunostaining to identify TH+ cells in the region of SN (Figure 4), which confirmed the microscopic evaluation. The prolonged treatment with ROT resulted in a profound loss of TH+ neurons in comparison with Control, while administration of pomegranate juice improved the neuron survival. Treatment with pomegranate juice alone did not affect TH+ cells’ survival.

### 2.5. Biochemical Examinations

#### 2.5.1. Oxidative Stress Markers and Mitochondrial Aldehyde Dehydrogenase Activity

**Figure 5:**
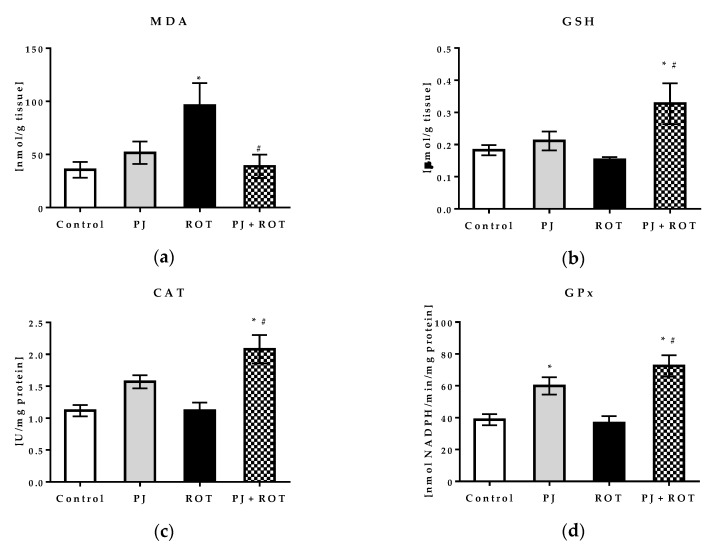

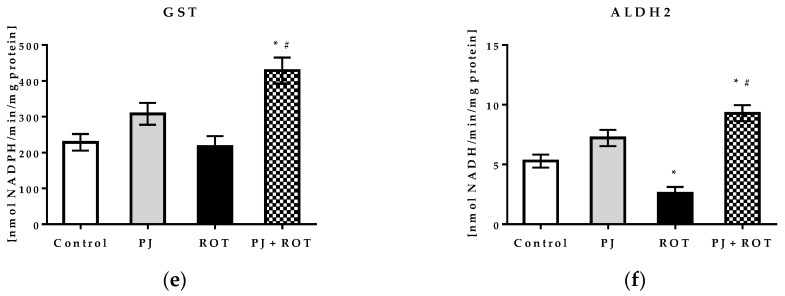
Effect of pomegranate juice treatment (PJ) on: (**a**) Malondialdehyde (MDA) concentration; (**b**) reduced glutathione (GSH) concentration; (**c**) catalase (CAT) activity; (**d**) glutathione peroxidase (GPx) activity; (**e**) glutathione S-transferase (GST) activity; (**f**) mitochondrial aldehyde dehydrogenase 2 (ALDH2) activity, in the midbrain homogenate of rotenone (ROT) injected rats. Data are presented as mean values ±SEM of eight rats per group and analyzed using one-way ANOVA followed by Sidak’s multiple comparisons test. * *p* < 0.05 vs. Control. # *p* < 0.05 vs. ROT.

To assess the effects of pomegranate juice treatment against oxidative stress, malondialdehyde (MDA) as the measure of lipid peroxidation and markers of the endogenous antioxidant system, including reduced glutathione (GSH) and antioxidant enzymes activity, were assayed in the midbrain. In addition, we assessed the activity of mitochondrial aldehyde dehydrogenase (ALDH2), the enzyme protecting against oxidative stress by detoxification of cytotoxic aldehydes, since we triggered mitochondria-mediated oxidative stress by rotenone [27]. Rotenone administration induced a significant 170% rise in the MDA level in comparison with control rats (Figure 5a). Consistently, mitochondrial ALDH2 activity was decreased by 51% (Figure 5f). Pomegranate juice administration to the rotenone challenged animals attenuated the level of lipid peroxidation by 60%, which was similar to that in the control group. The inhibition of lipid peroxidation correlated with about a 2.5-fold increase, even above the control level, in mitochondrial ALDH2 activity. The response of the endogenous antioxidant system in experimental groups was diversified. The activities of catalase (CAT), glutathione peroxidase (GPx), and glutathione S-transferase (GST) in the ROT group were slightly decreased, however their values did not differ significantly from Control. Administration of PJ to ROT-injected animals increased the activities of CAT, GPx, and GST by 85%, 98%, and 97%, respectively, as compared to those observed in the ROT group (Figure 5c–e), and they were even higher than those in the control group. Treatment with PJ alone also caused an enhancement in GPx activity by 55% and in CAT activity by 41% vs. the control value, although the latter change was not statistically significant. The GSH level was slightly (by 16%), but not significantly, decreased in rats administered with ROT, while combined treatment with PJ and ROT caused its increase by 114% (Figure 5b). The activity of SOD was not statistically different among the groups (data not shown herein).

#### 2.5.2. Inflammation Markers

**Figure 6:**
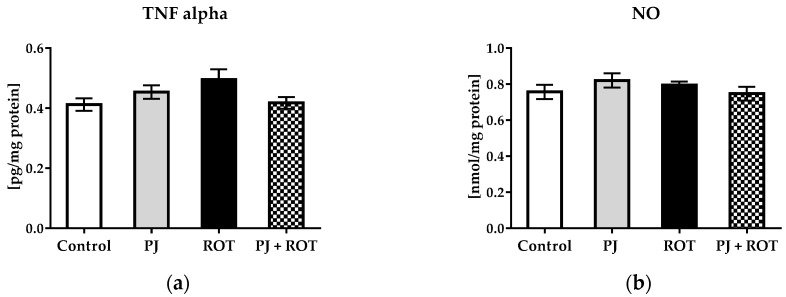
Effect of pomegranate juice treatment (PJ) on: (**a**) Tumor necrosis factor (TNF-α) expression; (**b**) total nitrite concentration, in the midbrain of rotenone (ROT) injected rats. Data are presented as mean values ±SEM of eight rats per group and analyzed using one-way ANOVA followed by Sidak’s multiple comparisons test.

The expression of tumor necrosis factor-alpha (TNF-α) (Figure 6a) and nitrites concentration (Figure 6b) were similar across all groups with no statistically significant differences.

### 2.6. Apoptosis Markers

**Figure 7:**
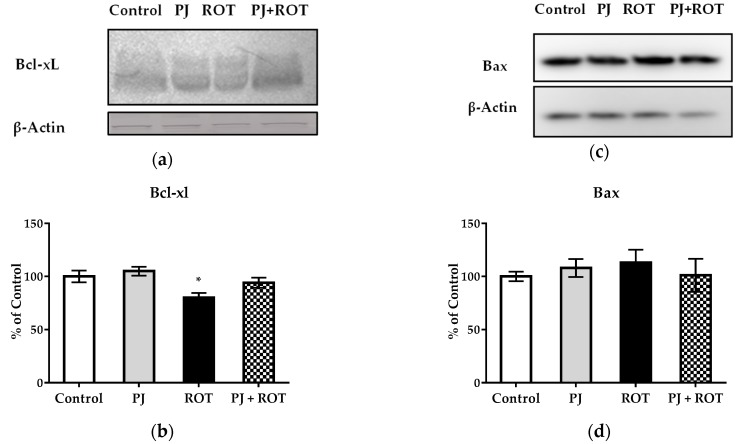
Effect of pomegranate juice treatment (PJ) on: B-cell lymphoma-extra-large (Bcl-xL) expression shown as representative immunoblots (**a**), % of control value ±SEM of eight rats per group (**b**), apoptosis regulator Bax expression shown as representative immunoblots (**c**), % of control value ±SEM of eight rats per group (**d**) in the midbrain of rotenone (ROT) injected rats. The results are presented as relative levels normalized to β-Actin, which was used as an internal control. Data analyzed using one-way ANOVA followed by Sidak’s multiple comparisons test. * *p* < 0.05 vs. control.

Rotenone caused a 20% decrease in the expression of pro-survival protein Bcl-xL, as compared with the control values. PJ treatment of the ROT challenged animals restored its expression by 18% (not significantly) almost to the level measured in the control rats. The expression of the second assayed Bcl-2 family member—apoptosis regulator Bax—was not affected, neither by pomegranate juice nor rotenone administration individually or combined (Figure 7 and Figure S2).

### 2.7. α-Synuclein Expression

**Figure 8:**
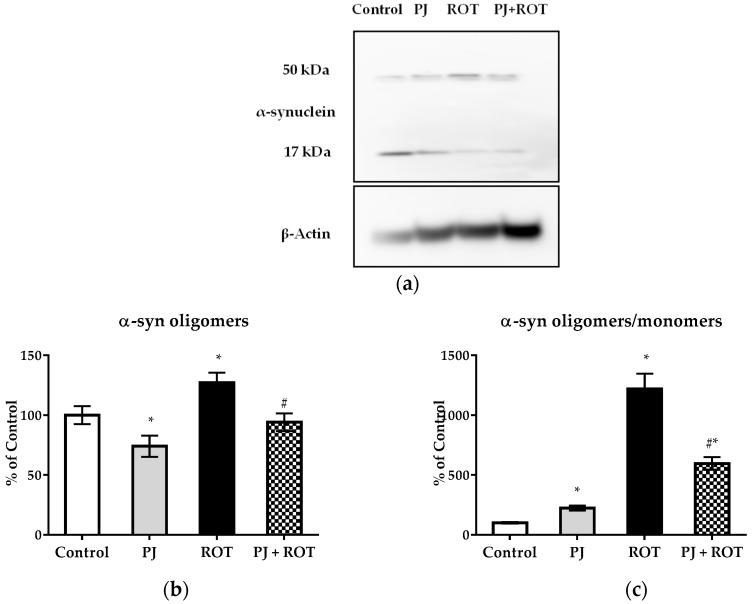
Effect of pomegranate juice treatment (PJ) on levels of the 17 kDa isoform of α-synuclein (monomers) and 50 kDa isoform of α-synuclein (oligomers) shown as representative immunoblots (**a**), on the level of α-synuclein oligomers (**b**) and on the ratio of α-synuclein oligomers/monomers (**c**) shown as % of control value ±SEM of eight rats per group in the midbrain of rotenone (ROT) injected rats. The results are presented as relative levels normalized to β-Actin, which was used as an internal control. Data analyzed using one-way ANOVA followed by Sidak’s multiple comparisons test. * *p* < 0.05 vs. Control; # *p* < 0.05 vs. ROT group.

To further confirm the observation that PJ treatment can protect neurons, we determined the level of α-synuclein, responsible for deleterious impact when abnormally accumulated in neurons, widely regarded as a pathological hallmark of PD [25]. Western blot analysis revealed that ROT caused an 27% increase in level of α-synuclein oligomeric species, which corresponded to 11-fold increase in the ratio of α-synuclein oligomers/monomers in the midbrain compared to that of control rats. The alteration was significantly ameliorated by PJ treatment in rats challenged with ROT as there was no significant difference in oligomeric fraction of this protein between PJ + ROT and control groups (Figure 8).

### 2.8. Urolithin a Determination

**Figure 9:**
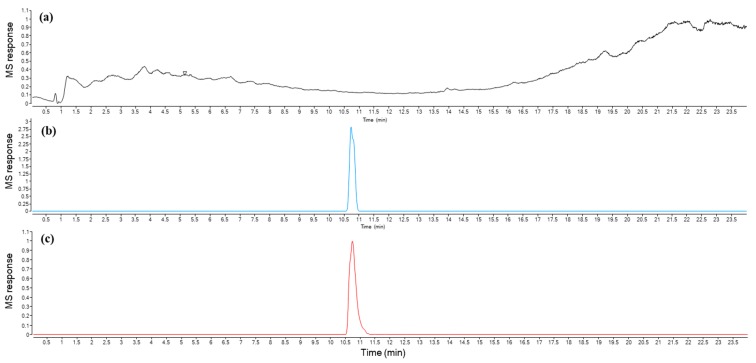
Representative UPLC-UV-QTOF chromatograms (**a**) total ion chromatogram for a brain sample of a rat treated with PJ; (**b**) selected ion chromatogram of *m*/*z* 227.0354 for a brain sample of a rat treated with PJ; (**c**) selected ion chromatogram of *m*/*z* 227.0354 for pure UA standard.

In order to examine whether urolithin A (UA) could be a neurologically active metabolite of pomegranate juice able to contribute to the overall neuroprotective effects observed in a rotenone model of Parkinson’s disease, we checked whether it is distributed into the brain. Therefore, we evaluated the concentration of UA in plasma and its deposition in the brain. The detection of UA in brain and plasma samples was performed by high-resolution UPLC-ESI-QTOF-MS. Even if the total ion chromatograms were too complex to allow the detection of UA (Figure 9a), the high selectivity and sensibility given by this mass spectrometer allow the detection and identification of UA in the brain and plasma samples treated with pomegranate juice by using the selected ion chromatograms for the UA high resolution mass in negative mode *m*/*z*: 227.0344 for [C_13_H_8_O_4_-H]- (Figure 9b). This peak was identified and confirmed to be UA by comparison with its UPLC retention times and mass spectra of an authentic chemical standard (Figure 9c). Moreover, its identity was confirmed by the determination of its molecular formula by HRESIMS technique from the detected ion at *m*/*z* 227.0354 [M − H]^−^ (*m*/*z* calcd 227.0344). The concentration of UA in the brain was 1.68 ± 0.25 ng/g tissue and in plasma 18.75 ± 3.21 ng/mL.

## 3. Discussion

Parkinson’s disease itself is not considered fatal [28]; however, due to a lack of any disease-modifying therapy, as disease progresses, swallowing turns to be compromised causing aspiration pneumonia that can life threatening [29]. Moreover, since the quality of life of PD patients is significantly diminished, there is an ongoing search for compounds capable of protecting neurons from a broad range of insults. As we previously reviewed [1] the number of studies supports the generally agreed view that polyphenols and/or their metabolites contribute to the neuroprotective effects of plant-derived preparations, which appear to be promising neuroprotective agents. With regard to pomegranate, as mentioned earlier, there is a research bias away from PD. Studies on the neuroprotective properties of pomegranate have mainly focused on AD in animal models [9,10,11,12,13,14]. To the best of our knowledge, only a few studies were performed using a PD model [22,23]. The study presented herein aimed to call into question whether pomegranate juice may provide neuroprotection against PD. For this purpose, we applied the environmentally relevant rotenone model of PD. Rotenone, an inhibitor of respiratory complex I, disrupts mitochondrial electron transport and generates ROS and due to the high lipophilicity, it easily crosses biological membranes, including the blood–brain barrier. Rats exposed to prolonged, low-dose rotenone treatment develop selective degeneration of nigral dopaminergic neurons with histopathological hallmarks of PD and PD-like locomotor symptoms due to sustained inhibition of complex I and related oxidative injury in the brain [26]. Since bodyweight (b.w.) loss in animal rotenone models of PD has been demonstrated along with behavioral deficits, a loss of tyrosine hydroxylase (TH+) positive neurons of the SN, an increased apoptosis and a decreased antioxidant defense in the midbrain [30,31,32] we also monitored this parameter. In our study, exposure of rats to rotenone caused a significant decrease in b.w. gain in the last three weeks of the experiment that is in line with findings by Binienda et al. [33]. The beneficial effect of pomegranate juice treatment against weight loss was noticed one week later.

As mentioned above, rotenone is known to produce PD-like behavioral features, which in our experiment were manifested as impaired postural stability. Pomegranate juice treatment attenuated the rotenone-induced behavioral deficit. This is in agreement with a finding that chronic pomegranate juice co-administration improved movements and reduced levodopa-induced dyskinesia in an MPTP mice model of PD [23]. Our findings share some similarities with the findings for other polyphenols. Curcumin administered for 50 days and piceid for five weeks also have been reported to improve rotenone-induced postural defects [34,35].

Given the fact that loss of dopaminergic neurons triggers deregulation of motor symptoms [36], which we had previously demonstrated in an inducible transgenic PD model [37], we performed both the microscopic examination and the immunofluorescent analysis of TH+ neurons in sections of the SN area, which is the region of interest in experimental PD models due to the vulnerability of dopaminergic neurons in the brain [38]. In agreement with the other authors’ findings [39,40], a microscopic examination proved that rotenone-induced neurotoxicity involved the midbrain, while sections of cerebellum and cortex showed a quite normal structure. H&E staining revealed degenerative and necrotic changes in the form of shrunken neurons with dark cytoplasm and pyknotic nuclei. The neurodegeneration was reflected by the loss of TH-positive cells in the SN region, which is in line with previous studies [41,42]. The administration of pomegranate juice to rotenone-intoxicated rats ameliorated the damaging effect of the neurotoxin since only some neurons with dark cytoplasm were still observed in the SN region and enhanced survival of TH+ neurons in this area was noticed. This finding is in agreement with the previously reported lower neuronal loss observed in rats with I/R injury pretreated with punicalagin [43]. Interestingly, the systemic administration of its metabolite UA has protected mice against ischemic brain injury that correlated with an improved neurological deficit score [44]. Based on this, it seems likely that urolithin A could contribute to the protective effect of PJ-treatment against rotenone-induced neuronal degeneration which results in behavioral improvement.

The preferential degeneration of midbrain neurons, especially in SNpc region, compared to other nearby catecholaminergic neurons, is due to tremendous oxidative stress associated with high dopamine turnover rates. Its reactive aldehyde metabolites, mainly 3,4-dihydroxyphenylacetaldehyde, have been demonstrated to contribute to the pathogenesis of PD. The autoxidation of dopamine also contributes to increased generation of detrimental ROS, which, via lipid peroxidation, leads to the production of other reactive aldehydes, such as MDA, and consequently causes degeneration of dopaminergic neurons [24]. In this study and in agreement with the previous works [39,40], rotenone selectively affected the midbrain area, where the increased level of MDA was detected. Pomegranate juice treatment provided substantial protection against rotenone-induced lipid peroxidation, which probably was assured by increased activity of mitochondrial ALDH2—the principal enzyme involved in detoxifying aldehydes. ALDH2 converts MDA and other ROS-induced aldehydes to less toxic acid products and is highly expressed in the brain, especially in the dopaminergic neurons of the midbrain. Inhibition of ALDH2 by some pesticides in turn leads to the accumulation of reactive aldehydes, preferential degeneration of dopaminergic neurons, and the development of PD [27]. Consistent with this idea, Chiu et al. [27] have shown that administration of a pharmacological activator of ALDH2 reduced the rotenone-induced accumulation of the lipid peroxidation endproduct—4-hydroxynonenal in the SN—and prevented loss of dopaminergic neurons as a result. A decline of neurological deficit score and brain cell loss [45] in a homocysteine rat model of AD and in a model of cerebral I/R injury in the rat was attributed to the clearance of reactive metabolites by ALDH2. In line with this, resveratrol supplementation preserving cortical ALDH2 level provided substantial protection against oxidative stress-mediated neocortex damage in high-fat/sucrose (HFS)-fed rhesus monkey [46].

On the other hand, the neuronal population is particularly susceptible to oxidative damage since it has a relatively low antioxidant capacity [24]. Therefore, we assessed the effects of pomegranate juice treatment on the antioxidant defense system in the midbrain. Although many authors have shown the decrease in GSH level and/or antioxidant enzymes activity in this brain area of rats exposed to rotenone [31,40,41,42,47,48,49,50], in our experiment, these markers were not affected significantly by rotenone administration. Differences in the response can be associated with the higher doses of rotenone, ranging between 1.5 mg/kg b.w. and 2.5 mg/kg b.w., and a route of its administration, which was mainly intraperitoneal [39,40,41,42]. Manjunath and Muralidhara [51] have used lower rotenone dose, i.e., 1 mg/kg b.w., and noticed no significant change either in SOD or in GPx activity in the striatum of rats. The authors even have reported an increase in striatal SOD and CAT activity of mice injected (i.p.) with rotenone in a dose of 0.5 mg/kg b.w. [51]. In our experiment, the activity of antioxidant enzymes increased in response to combined treatment (ROT+PJ) with the exception of SOD activity (data not shown herein). Accumulating evidence supports the inducing effect of pomegranate [7,52,53] and its active compound punicalagin [43,54] on the endogenous antioxidant system in different experimental models. We demonstrated a trend toward enhancing the endogenous antioxidant system following treatment with pomegranate juice alone; the increase was significant only for GPx activity. It is very likely that ellagitannins present in pomegranate juice contributed to this effect since increased activity and expression of antioxidant enzymes, including GPx activity, have been reported in the brain of rodents treated with punicalagin and berry-derived ellagitannin-enriched fractions, respectively [54,55]. Sun et al. [56] identified the AMPK-nuclear factor-erythroid 2 p45-related factor 2 (Nrf2) pathway as a mechanism contributing to the enhancement of the antioxidant defense system in the hypothalamus of hypertensive rats treated with pomegranate extract [56].

We did not find any significant difference in inflammatory response among the groups, but it might be related to the moderate level of rotenone-induced lipid peroxidation. Several authors have reported an increased expression of proinflammatory cytokines, including TNF-α and/or level of total NO accompanied by very high (3–5-fold) increase in MDA level in the midbrain of animals administered with rotenone [42,50,57].

Oxidative stress is suggested to initiate apoptotic neuronal cell death in PD. Neurotoxins such as rotenone caused cell death through the modulation of members of the B cell lymphoma 2 family of proteins (Bcl-2) [58]. The balance between antagonistic family members such as apoptosis inhibitor Bcl-xL and its promotor Bax plays a key role in determining cell survival or death [59]. Dhanalakshmi et al. [60] who observed a substantial (about 4.5-fold) increase in lipid peroxidation in the striatum of rats treated with rotenone (2.5 mg/kg/day, i.p., for 45 days) have reported a significant rise in striatal Bax level. In our study, chronic rotenone treatment significantly decreased only pro-survival Bcl-xL expression. Pomegranate juice administration ensured maintaining its amount at the control level and the rescue of midbrain neurons from rotenone toxicity was observed. The result shares a similarity with Bernier et al.’s [46] findings showing that resveratrol supplementation may overcome HFS-induced neocortex damage by protecting the expression of the anti-apoptotic Bcl-2 and ALDH2 proteins in a nonhuman primate.

Moreover, along with the neuronal loss in the SN [61,62] and motor impairment [61], the accumulation of α-synuclein, a hallmark of PD, in rats exposed to chronic subcutaneous low doses of rotenone has been recently reported [61,62]. α-Synuclein accumulation is thought to underlie the neurodegeneration in PD. The pathogenicity of this protein is attributed to the transition from a native α-helical conformation to a β-sheet structure that polymerizes to toxic forms [25]. We found that the rotenone-induced α-synuclein aggregation was significantly diminished by PJ treatment, as the level of its early oligomeric species was similar to that in the control group. It seems that PJ caused down-regulation of α-synuclein protein expression since in rats treated alone with the juice, the levels of α-synuclein oligomers was decreased while the ratio of this fraction to monomeric was higher than in Control. This effect is likely to be involved in the improvement of neuronal cell survival, which was also reported for other natural preparations [42,62]. 

Because treatment with pomegranate juice protected rats against rotenone-induced motor deficit, neuron degeneration, lipid peroxidation, α-synuclein aggregation, and inhibition of mitochondrial ALDH2 activity in the midbrain, as well as caused the maintenance of pro-survival Bcl-xL expression at the control level, we surmised that the generated in vivo ellagitannin-derived metabolite, urolithin A, might contribute to this effect. We, therefore, sought to determine its presence in plasma and in the brain. Our findings provide, to the best of our knowledge, the first evidence that urolithin A is distributed to the brain after the intake of pomegranate juice. Seeram et al. [63] have reported the presence of urolithin A, at a similar level as that observed in our study, in plasma and brain tissue of mice that received synthesized UA (0.3 mg/mouse) by the oral and intraperitoneal route; however, in mice, following pomegranate extract administration, UA was detected neither in the plasma nor brain. Based on available data about its activity, it could be suggested that it contributed to the overall neuroprotective effects demonstrated in our study; however, further studies are required to confirm this assumption.

The results of this study indicate that treatment with pomegranate juice prevents PD-like features in rats. Its efficiency in suppressing lipid peroxidation correlated with the enhanced activity of mitochondrial ALDH2 and normalization of the expression of anti-apoptotic Bcl-xL protein. The histological analysis demonstrated a substantially lower number of degenerated neurons in the SN as a result of pomegranate administration to rats challenged with rotenone. In addition, our study provides considerable insight into the neuroprotective potential of pomegranate ellagitannins-derived metabolite—urolithin A. However, this study is the first step towards enhancing our understanding of the capability of pomegranate for prevention of Parkinson’s disease and the mechanistic research involving mitochondria-related processes and a metabolomic approach as well as further studies in others PD models should be undertaken.

## 4. Materials and Methods

### 4.1. Pomegranate Juice

Commercial 6-fold concentrated pomegranate juice (PJ) was obtained from Alter Medica (Żywiec, Poland). The product was manufactured in accordance with the principle of HACCP (hazard analysis and critical control point) and fruit ingredients are fully compliant with the Code of Practice of the European Fruit juice Association (AIJN). Since pomegranate’s ellagitannins and their hydrolysis product—ellagic acid—have been demonstrated to be precursors of potentially neuroprotective urolithins, including urolithin A [8], which we detected in this study, we identified these phenolics in the tested juice. The ellagitannin composition of PJ was as follows: Galloyl-hexoside, ellagic acid-hexoside, 3-bis-HHDP-hexoside (pedunculagin), 4-galloyl-bis-HHDP-hexoside (casuarinin), and ellagic acid (Figure S1). The total polyphenols content expressed as g of ellagic acid (EA) equivalents per L of juice was 18.90 ± 0.96 g/L. The ellagitannin identification was performed according to the protocol described previously by Oszmianski et al. [64] using a Waters Acquity Ultra Performance LC system composed of an autosampler, binary solvent manager, and photodiode array detector (PDA; Waters Corporation, Milford, MA, USA). The system was coupled to a quadrupole time-of-flight mass spectrometer (Waters, Manchester, UK) equipped with an electrospray ionization (ESI) source operating in negative and positive ion modes.

### 4.2. Animals

The animal experiment was performed on six-week old male albino Wistar rats weighing 250–300 g. All the animals used in this study were bred in the Department of Toxicology, Poznan University of Medical Sciences (Poznań, Poland). Animals were held (four rats/cage) in polycarbonate cages (Tecniplast, Buguggiate, Italy) with wood shavings in a room maintained under 12 h light/dark cycle, 22 ± 2 °C, 40–54% relative humidity, and controlled circulation of air. A commercial diet (ISO 22000 certified laboratory feed Labofeed H) and drinking water were available ad libitum.

### 4.3. Experimental Design

**Figure 10:**
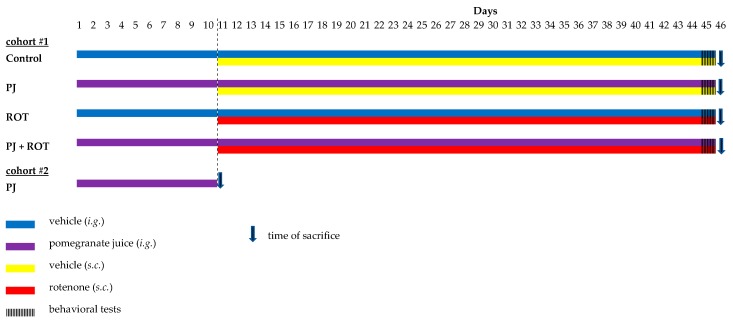
Schematic representation of the experimental design used in the study.

In order to induce PD in rats, rotenone (ROT, Sigma-Aldrich, Poznań, Poland) was injected subcutaneously once daily for 35 days in a dose of 1.3 mg/kg body weight. The doses and schedule of ROT used in the present study were established based on our results from preliminary studies, and they are similar to those previously described in published reports [33,39,57,65] with slight modifications (Figure 10). Forty rats (cohort #1) were divided randomly into four groups, with 10 animals in each. Group I: Rats receiving a vehicle, designated as a control group (Control). Group II: Rats treated with pomegranate juice alone in a dose of 500 mg/kg b.w./day (i.g.), designated as pomegranate juice-treated group (PJ). Group III: Rats injected with rotenone (1.3 mg/kg b.w./day, s.c.) alone from the 11th day of the experiment, designated as rotenone group (ROT). Group IV: Rats treated with pomegranate juice in a dose of 500 mg/kg b.w./day (i.g.) and injected with rotenone from the 11th day, designated as pomegranate juice + rotenone group (PJ + ROT). The experiment lasted a total of 45 days, including 10 days pre-treatment with PJ and 35 days combined treatment with PJ and ROT. The animals were observed daily for clinical signs of toxicity, and bodyweight was recorded weekly. Twenty-four hours after the last treatment, the rats were euthanized with ketamine/xylazine (100 U/7.5 mg/kg b.w., intraperitoneally) and perfused intracardially with isotonic sodium chloride solution. Following perfusion, the brain was removed quickly, the midbrain, cortex, and cerebellum were separated on ice, and the tissues were snap frozen using dry ice and stored at −80 °C until further use. For the purpose of the microscopic examination the brains of two rats from each group, were harvested after intracardial perfusion with isotonic sodium chloride solution, followed by 4% (*w*/*v*) paraformaldehyde in a 0.1 M sodium phosphate buffer, pH = 7.4 (Merck, Warszawa, Poland). The brains were then fixed with the buffered paraformaldehyde at 4 °C with gentle shaking for 24 h and subsequently exchanged with graded ethanol three times a day for three consecutive days at 4 °C prior to cryostat sectioning.

In order to examine the distribution of urolithin A into the brain, 3 rats (cohort #2) were treated with pomegranate juice in a dose of 500 mg/kg/b.w./day alone for 10 days. The rats were anesthetized 6 h after last treatment with ketamine/xylazine (100 U/7.5 mg/kg b.w., intraperitoneally) and blood was withdrawn from the heart to the heparinized tubes. The brain was harvested after whole body perfusion with phosphate buffered saline, pH 7.4, to avoid overlapping of metabolites from the residual blood.

Experiments were performed in accordance with Polish governmental regulations (Dz. U. 05.33.289) and with EU Directive 2010/63/EU for animal experiments. The study protocol was approved by the Local Ethics Committee on the Use of Laboratory Animals in Poznan, Poland (63/2015, 4 Sep 2015 and 14/2018, 27 Apr 2018).

### 4.4. Postural Instability Test

This test was performed according to Cannon et al. [30] method. Each animal was held vertically, and one forelimb was allowed to contact the table lined with sandpaper. The rat’s center of gravity was then advanced and pushed forward until the rat initiated a step. The displacement distance required for the rat to regain the center of gravity was recorded. Three trials for each forelimb were recorded and the average distance was reported.

### 4.5. Hematoxylin and Eosin (H&E) Staining

Histological examinations were performed in INFO-PAT laboratory, Poznań, Poland. Following fixation described in Section 2.3, brains were embedded in paraffin, and sliced into 4 μm coronal sections and stained with hematoxylin and eosin at 22–24 °C. The slides were evaluated by light microscopy (BX61VS, Olympus, Tokyo, Japan), and scanned using a digital camera (HVF22CL 3CCD, Hitachi, Tokyo, Japan) and the Panoramic Viewer software (3DHISTECH, Budapest, Hungary).

### 4.6. Immunofluorescent Staining of TH+ Neurons

Paraformaldehyde-fixed rat brains embedded in paraffin as in 4.5 were cut into coronal sections on rotary microtome at 7 µm thickness. Chosen sections from corresponding regions of midbrain were deparaffinized in xylene, boiled in citrate buffer in a microwave oven for antigen retrieval, and blocked with 5% normal pig serum (NPS, Vector Laboratories, Burlingame, CA, USA) for 30 min. For immunofluorescent staining, sections were subsequently incubated with the anti-tyrosine hydroxylase antibody (sheep, 1:500, AB1542, Millipore, Temecula, CA, USA) in 5% NPS overnight in +4 °C. Afterwards, sections were rinsed and incubated with secondary anti-sheep Alexa-488 secondary antibody (1:100, Invitrogen, Carlsbad, CA, USA) in PBS for 30 min and mounted with Vectashield Hard Set Mounting Media with DAPI (Vector Laboratories, Burlingame, CA, USA). Stained sections were imaged and manually analyzed under a fluorescent microscope Eclipse50i (Nikon, Tokyo, Japan) equipped with a digital camera and digitalized by NIS Elements software.

### 4.7. Biochemical Examinations

Frozen brain tissues were homogenized with a lysis buffer (Cell Lysis Buffer 2; Bio-Techne-R&D Systems, Minneapolis, MN, USA) supplemented with a cocktail of protease and phosphatase inhibitor (Protease Inhibitor Cocktail I, Bio-Techne-Tocris, Minneapolis, MN, USA) at a weight:volume ratio of 1:2, using a handheld tissue homogenizer. The homogenate of each sample was centrifuged at 10,000 *g* for 20 min at 4 °C. The supernatant was collected for the biochemical assays with the exception of mitochondrial ALDH2 activity assay.

#### 4.7.1. Lipid Peroxidation

Lipid peroxidation was determined by the reaction of its end product malondialdehyde (MDA) with thiobarbituric acid (TBA) according to the manufacturer’s protocol provided with the lipid peroxidation (MDA) assay kit (Sigma-Aldrich, Poznań, Poland).

#### 4.7.2. Endogenous Antioxidants

The reduced glutathione (GSH) level and antioxidant enzymes activities were determined spectrophotometrically as previously described [66]. Briefly, GSH was quantified with the Ellman’s reagent. The superoxide dismutase (SOD) activity was measured using spontaneous epinephrine oxidation. The catalase (CAT) activity was assayed by the measurement of hydrogen peroxide reduction–oxidation. Glutathione peroxidase (GPx) activity was determined by measuring NADPH oxidation using hydrogen peroxide as a substrate. Glutathione S-transferase (GST) activity was determined using 1-chloro-2,4-dinitrobenzene (CDNB) as a substrate.

#### 4.7.3. Mitochondrial Aldehyde Dehydrogenase (ALDH2) Activity

The activity of this enzyme was determined using the ALDH2 activity assay kit according to the manufacturer’s protocol (ab115348, Abcam). Tissue samples of the brain were homogenized in three-volumes of ice-cold phosphate-buffered saline. The homogenate was treated by adding an extraction Buffer to a sample protein concentration of 10 mg/mL and centrifuged at 16,000 × *g* 4 °C for 20 min followed by incubation on ice for 20 min. The samples in the volume of 100 μL were then subjected to a procedure of microplate assay.

#### 4.7.4. Tumor Necrosis Factor-Alpha (TNF-α)

To quantify tumor necrosis factor-alpha (TNF-α), 50 μL of brain homogenate supernatant (Section 4.7) was subjected to ELISA assay according to the manufacturer’s protocol, provided with the kits (RTA00, Bio-Techne-R&D Systems, USA).

#### 4.7.5. Nitrite Concentration

Nitrite is formed by the spontaneous oxidation of nitric oxide (NO) under physiological conditions. As a measure of NO, we determined the nitrite concentration spectrophotometrically with the use of the Griess diazotization reaction, according to Gilchrist et al. [67]. Briefly, equal volumes of brain homogenate (Section 4.6) and Griess reagent (1% sulfanilamine, 0.1% *N*-(1-naphyl)-ethylene-diamine dihydrochloride, 2.5% H_3_PO_4_) were mixed. The absorbance at 540 nm was measured and the nitrite concentration was calculated from a sodium nitrite (NaNO_2_) solution standard curve.

#### 4.7.6. Protein Determination

The quantity of protein in samples was measured employing the Bicinchoninic Acid Protein Assay Kit following the manufacturer’s instruction (BCA1 AND B9643, Sigma-Aldrich, Poznań, Poland).

### 4.8. Western Blotting

For the determination of the Bax, Bcl-xL, and α-synuclein protein levels, Western blot analysis was performed. The samples containing 5 µg (100 µg for Bcl-xL) of proteins were separated on 10% or 12% SDS-PAGE gels and transferred to nitrocellulose membranes. After blocking with 10% skimmed milk, the proteins were probed with rabbit Bax, mouse Bcl-xL, rabbit β-actin (Santa Cruz, CA, USA), and rabbit α-synuclein (Cell Signaling Technology) antibodies. The Western blotting detection system and SDS-PAGE Gels (10%, 12%) were purchased from Bio-Rad Laboratories (Hercules, CA, USA). As the secondary antibodies, the alkaline phosphatase-labelled anti-mose IgG (Santa Cruz, CA, USA) or HRP-linked antibody (Cell Signaling Technology) were used. The β-actin protein was used as an internal control. The amount of immunoreactive product in each lane was determined by densitometric scanning using a BioRad GS710 Image Densitometer (BioRad Laboratories, Hercules, CA, USA). The values were calculated as relative absorbance units (RQ) per mg protein.

### 4.9. Urolithin a Determination

#### 4.9.1. Isolation

Plasma samples were extracted with ACN: formic acid (98:2, *v*/*v*) according to the procedure described by Núñez-Sánchez et al. [68]. Briefly, thawed plasma samples were mixed with the extraction mixture by vortexing and sonification and subsequently centrifuged using refrigerated centrifuge (Eppendorf 5810 R, Hauppauge, NY, USA). The supernatant was reduced by evaporation in the speed vacuum (Savant SPD1010, ThermoFisher, Waltham, MA, USA).

Brain samples were extracted with methanol:HCl (99.9:0.1 *v*/*v*) as described by Núñez-Sánchez et al. [68] following enzymatic hydrolysis of conjugated UA metabolites according to Seeram et al. [69] with some modifications. Briefly, each brain was thawed, homogenized with pure water using an IKA T25 BASIC instrument (Janke and Kunkel, Ika-Labortechnik, Staufen, Germany), mixed with 500 units of β-Dglucuronidase (Sigma-Aldrich, Poznań, Poland) and 4 units of sulfatase (Sigma-Aldrich, Poznań, Poland) vortexed, sonicated, and then incubated at 37 °C for 45 min. After the incubation, samples were once again vortexed, sonicated, and subsequently centrifuged (Eppendorf 5810 R, US). The supernatant was reduced by evaporation in the speed vacuum (Savant SPD1010, ThermoFisher, Waltham, MA, US).

The dried samples were re-suspended in methanol and filtered through a 0.45 μm PVDF filter (Merc, Warszawa, Poland) before analysis by UPLC-ESI-QTOF-MS.

#### 4.9.2. UPLC-ESI-QTOF-MS Analysis

The UPLC-ESI-QTOF-MS system used was an Agilent 1290 Infinity (Agilent, Les Ulis, France) equipped with an ESI-QTOF-MS (Agilent 6530 Accurate Mass, Agilent, Les Ulis, France). Chromatographic separation was carried out on an Eclipse Plus C18 column (2.1 × 100 mm, 1.8 µm, Agilent, Les Ulis, France). The solvents used were water with 0.1% formic acid for solvent A and methanol with 0.1% formic acid for solvent B with the flow rate of 0.3 mL/min. The gradient of solvent B for was as follows: Stayed at 5% during 0.5 min; 5% to 15% in 2.5 min; 15% to 30% in 8 min; 30% to 50% in 4 min; 50% to 90% in 6 min; stayed at 90% during 4 min; and the UPLC column was equilibrated for 3 min. The gradient of solvent B for analysis was as follows: Stayed at 4% during 10 min; 4% to 95% in 4 min; stayed at 95% during 2 min; and the UPLC column was equilibrated for 3 min under the initial condition. ESI conditions were as follows: Gas temperature and flow were 300 °C and 9 L/min, respectively; sheath gas temperature and flow were 350 °C and 11 L/min, respectively; capillary voltage was 3500 V. The fragmentor was always set at 150 V. The data obtained were analyzed by MassHunter Qualitative Analysis software. A calibration curve was established using commercially available UA in the range of 1 ng/mL to 100 ng/mL.

### 4.10. Statistical Analyses

The results are presented as mean values ± SEM. The experiments were performed in duplicate and 8 animals per experimental group were used. For the analysis of urolithin A distribution, 3 animals were used. Comparisons between the control and ROT groups were performed by one-way analysis of variance (ANOVA) followed by Sidak’s multiple comparisons test. Differences were considered significant at *p* < 0.05. All statistical analyses and charts were performed using PRISM 6.0 software (GraphPad Software Inc., La Jolla, CA, USA).

## 5. Conclusions

In conclusion, the results provide evidence for the beneficial effect of pomegranate juice in rotenone-induced PD and suggest that ellagitannins-derived metabolite—urolithin A—may be a plausible active compound.

## Supplementary Materials

Supplementary materials can be found at https://www.mdpi.com/1422-0067/21/1/202/s1.

## Abbreviations

AD	Alzheimer disease
ALDH2	mitochondrial aldehyde dehydrogenase
APP	amyloid precursor protein
Bax	Bcl-2 family member - apoptosis regulator
Bcl-xl	B-cell lymphoma-extra large
CAT	catalase
EA	ellagic acid
ESI	electrospray ionization
H&E	hematoxylin and eosin
ETs	ellagitannins
H-I	hypoxic-ischemic
Iba1	ionized calcium-binding adapter molecule 1
I/R	ischemia-reperfusion
GSH	reduced glutathione
GST	glutathione S-transferase
HFS	high-fat/sucrose
LPO	lipid peroxidation
MDA	malondialdehyde
MPP^+^	1-methyl-4-phenylpyridinium
MPTP	4-phenyl-1, 2, 3, 6-tetrahydropyridine
NO	nitric oxide
NPS	normal Pig Serum
PD	Parkinson’s disease
PJ	pomegranate juice
ROS	reactive oxygen species
ROT	rotenone
SNpc	substantia nigra pars compacta
TH	tyrosine hydroxylase
TNF-α	tumor necrosis factor-alpha
UA	urolithin A
