# Supplementary material for: Neuroprotective Effects of Pomegranate Juice against Parkinson’s Disease and Presence of Ellagitannins-Derived Metabolite—Urolithin A—In the Brain
Source: Int J Mol Sci. 2019 Dec 27;21(1):202. doi: 10.3390/ijms21010202 (PMC6981883; doi:10.3390/ijms21010202)
Supplement: Supplementary file 1 [file ijms-21-00202-s001.pdf]

International Journal of
Molecular Sciences

MDPI

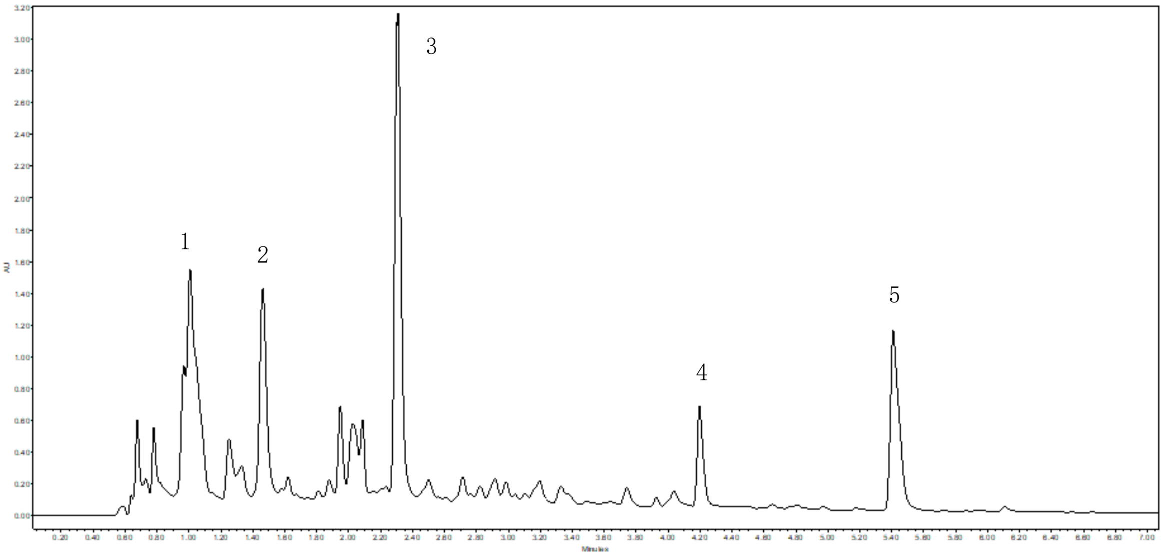

Figure S1. UPLC-PDA chromatogram at 250 nm of pomegranate juice (PJ). Proposed phenolic compounds were numbered by elution order 1-galloyl-hexoside, 2-ellagic acid-hexoside, 3-bis-HHDP-hexoside (pedunculagin), 4-galloyl-bis-HHDP-hexoside (casuarinin), 5- elagic acid.

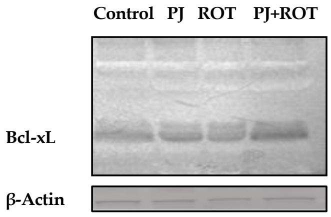

Figure S2. Uncropped full-length picture of Western blotting membrane presented in Figure 7a.
